# Transcatheter Embolization of a Rare Pulmonary Arterio-Arterial Malformation in a Nine-Year-Old Boy

**DOI:** 10.7759/cureus.97256

**Published:** 2025-11-19

**Authors:** Munazza Khanam, Nadia ElSayed, Saista Amin, Fatih Atik, Nader Francis

**Affiliations:** 1 Pediatric Medicine, Al Qassimi Women's and Children's Hospital, Sharjah, ARE; 2 Pediatric Gastroenterology, American Hospital Dubai, Dubai, ARE; 3 Pediatric Cardiology, Al Qassimi Women's and Children's Hospital, Sharjah, ARE; 4 Pediatric Pulmonology, Al Qassimi Women's and Children's Hospital, Sharjah, ARE

**Keywords:** acute hemoptysis, bronchoscopy, endoscopy, pulmonary arterial malformation, transcatheter ligation

## Abstract

Pulmonary arterio-arterial malformations exceptionally occur as rare vascular anomalies in the pediatric population, as they often challenge diagnoses because their symptoms are nonspecific. We report a previously healthy nine-year-old boy presenting with acute hematemesis initially suspected to be of gastrointestinal (GI) origin. Upper GI endoscopy and ear, nose, and throat (ENT) evaluation revealed normal findings; however, further investigations were indeed needed. Bronchoscopy, computed tomography (CT) angiography, and cardiac catheterization revealed a localized pulmonary hemorrhage because of an arterio-arterial malformation in the posterior basal segment of the right lung. The anomaly was successfully managed via transcatheter coil embolization. The management was successful. This case shows that thinking about uncommon lung blood vessel problems matters as you create possible diagnoses of child blood in the sputum or vomit, especially with early tests that are unclear. It also highlights the fact that multidisciplinary teams do critically collaborate to diagnose and then manage complex pediatric presentations.

## Introduction

Pulmonary vascular malformations are rare in the pediatric population and pose significant diagnostic and therapeutic challenges. Among these, arterio-arterial malformations within the pulmonary circulation are exceedingly rare, especially in children. These anomalies involve direct communication between two arteries, bypassing the capillary bed, which can lead to altered hemodynamics and bleeding complications [1].

Arterio-arterial malformations present with nonspecific respiratory symptoms, such as dyspnea or chest pain, or with alarming signs, like hemoptysis [2]. Hemoptysis in children is uncommon but potentially life-threatening. In the context of pulmonary vascular anomalies, it typically results from the rupture of fragile aberrant vessels [3]. Hematemesis, on the other hand, is a rare manifestation in such cases and may be due to swallowed blood from hemoptysis, especially in younger children who may not be able to expectorate effectively [4].

While much of the literature focuses on arteriovenous malformations, arterio-arterial malformations have been reported in both pediatric and adult populations, primarily in the cerebral, pulmonary, and coronary circulations. In adults, arterio-arterial malformations are more commonly seen in the context of atherosclerosis, trauma, iatrogenic injury, or prior vascular surgery. Known risk factors in pediatric patients include congenital vascular syndromes, in utero vascular developmental errors, or hereditary hemorrhagic telangiectasia (HHT). However, many cases are idiopathic and are identified only upon the development of clinical symptoms, such as bleeding, or during imaging [5].

To date, only a limited number of pediatric cases with isolated pulmonary arterio-arterial malformations have been reported [6]. This case contributes to the sparse literature and shows the importance of considering such rare vascular anomalies in the differential diagnosis of both gastrointestinal (GI) and respiratory bleeding in children. 

## Case presentation

A nine-year-old previously healthy boy was brought to the hospital by his parents due to one episode of vomiting frank blood. He had a sudden episode of hematemesis of around 20 ml of red blood after consuming a bag of spicy chips. No undigested food particles were present in the vomitus. The child denied any history of headache, abdominal pain, hematochezia, melena, epistaxis, hemarthrosis, or bruising. He had maintained good control of his asthma with a fluticasone inhaler for the past 2-3 years. His medical history was negative for any blood disorder, musculoskeletal abnormalities, or medication ingestion.

His family history was unremarkable. He was born to nonconsanguineous parents via normal vaginal delivery with no complications. He had never required any hospitalization thus far due to similar complaints or other issues.

Upon initial presentation to the hospital, the child had unstable vitals. His temperature was 36.7°C; however, his saturation on room air was 86%, and he maintained blood pressure within the 50th percentile. Initially, he was connected to 4 L oxygen by face mask, after which his saturation improved. The patient was alert and oriented. The ear, nose, and throat (ENT) exam was negative. He had no lymphadenopathy. Chest exam revealed good air entry with bilateral crepitations in the lower lung zones. The abdomen was soft and nontender, with no masses and a normal liver span, and the spleen was not palpable. A musculoskeletal exam and neurological examination were unremarkable.

While in the emergency room (ER), the child had one more episode of hematemesis, around 20 ml. He was given vitamin K and tranexamic acid and one dose of ceftriaxone. Laboratory investigations conducted at the time revealed a hemoglobin level of 11.6 g/dl. He was then transferred to Al Qassimi Women's and Children's Hospital for further management.

Upon arrival, the child was taken to the operating theater for an upper GI endoscopy due to the hematemesis as the initial impression.

**Figure 1:**
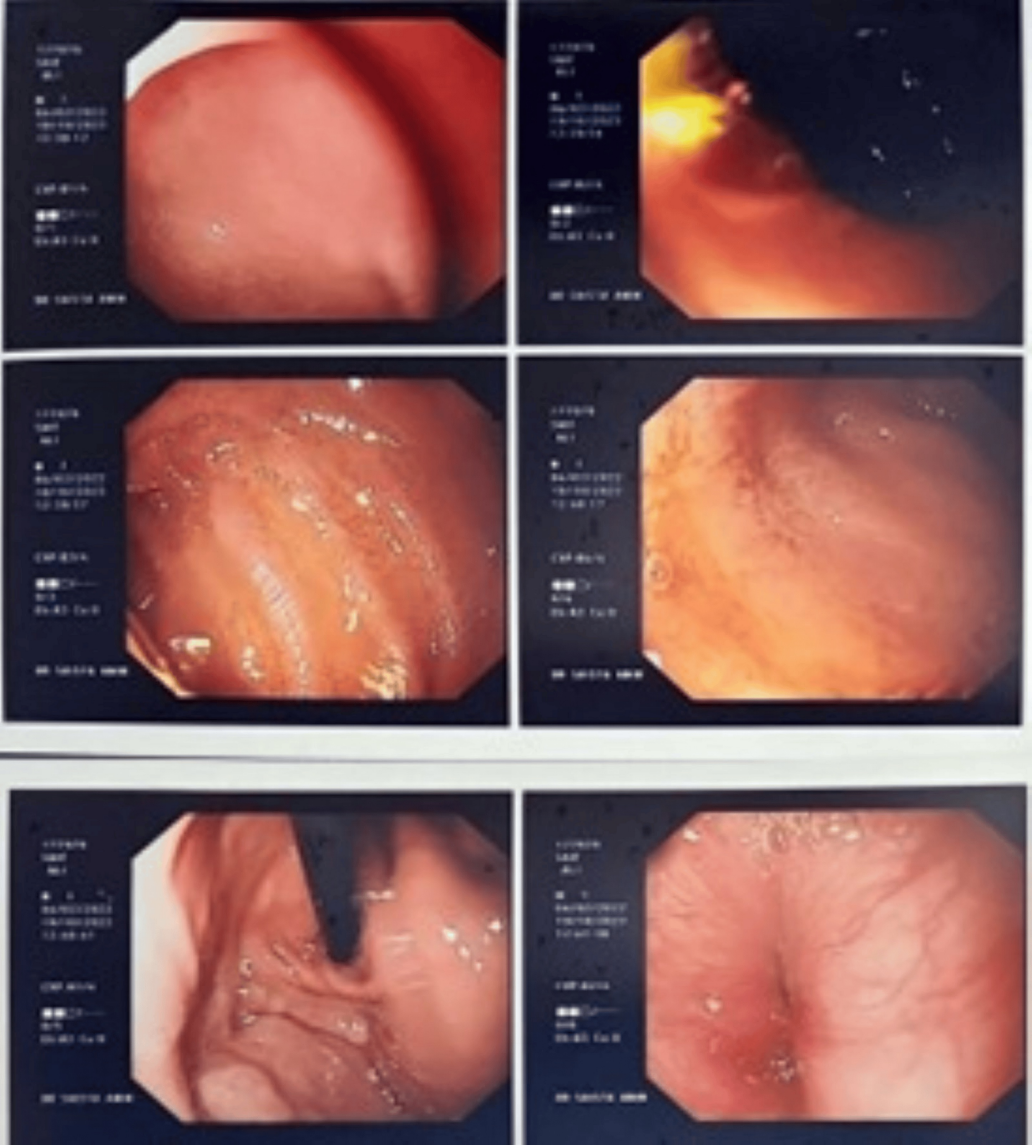
Upper GI endoscopy: the esophagus showed inflamed mucosa, with old blood clots that were noted in the stomach along with food particles. The rugosity of the stomach lining was maintained with no identified bleeding site or ulcers. D1 and D2 have normal mucosa. GI: gastrointestinal

The esophagus showed inflamed mucosa, with old blood clots that were noted in the stomach along with food particles. The rugosity of the stomach lining was maintained with no identified bleeding site or ulcers. D1 and D2 have normal mucosa (Figure 1). The rapid urease test was negative. A nasogastric (NG) tube was inserted, and the endoscopy was combined with biopsy samples from the antrum, D1, and D2. The NG tube was free of fresh blood after insertion. 

ENT was consulted for nasopharyngoscopy; no active focus of bleeding was detected in either the nostril, nasopharynx, hypopharynx, or larynx. The gums, hard palate, and oropharynx showed no signs of active bleeding.

The next afternoon, the child had an episode of frothy vomit from the mouth, around 100 ml, with fresh blood seen in the NG tube. During this time, his saturation dropped to 80% on room air, hence requiring oxygen supplementation, with a drop in hemoglobin from 12 g/dl to 10 g/dl.

**Figure 2:**
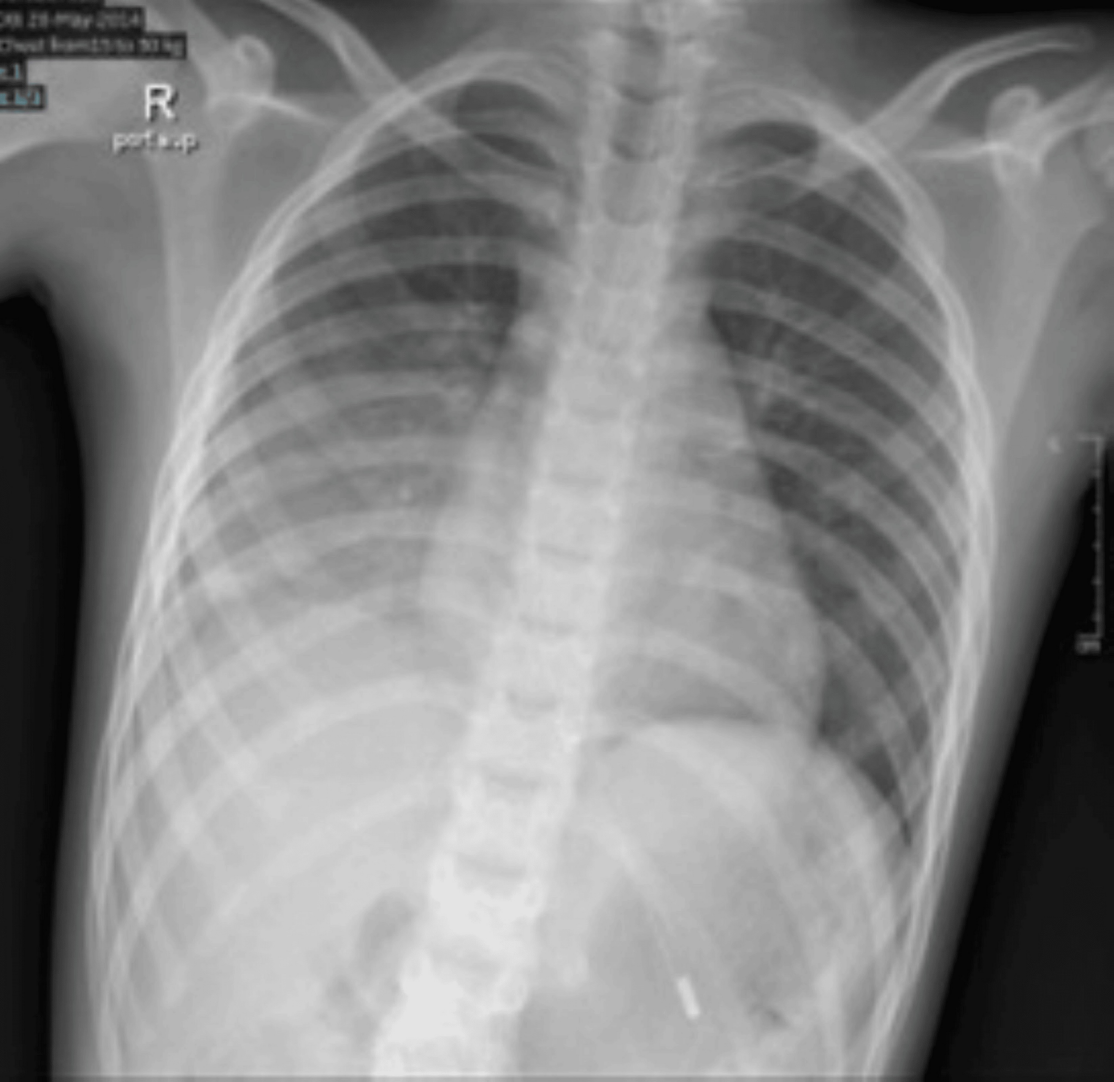
Chest X-ray showing homogeneous opacity in the right lower lung.

X-ray (XR) done at this time showed homogeneous opacification of the right lower lung. No lung ultrasound was done for this patient (Figure 2).

A pediatric pulmonologist was consulted, given this finding, and they concluded that there could be a possibility of pulmonary hemorrhage. It was concluded that more investigation would be needed to rule out or identify systemic causes of pulmonary hemorrhage, autoimmune, vasculitis, arteriovenous (AV) malformation, and foreign body and infection (tuberculosis (TB)). The following investigations were sent as part of the workup: cytoplasmic anti-neutrophil cytoplasmic antibody (C-ANCA) and perinuclear ANCA (P-ANCA), antinuclear antibody (ANA), anti-DNA antibody, rheumatoid factor, and anti-glomerular basement membrane (anti-GBM) antibody.

**Figure 3:**
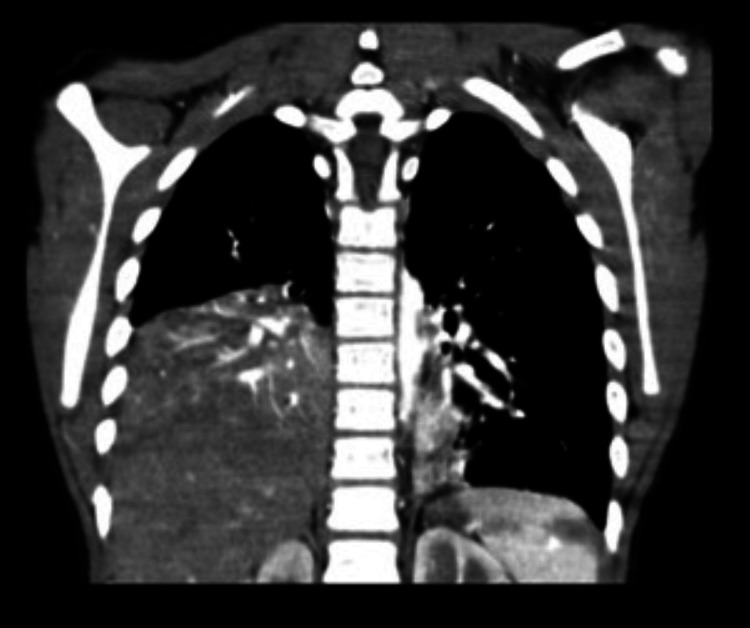
Chest CT showing bilateral posterior-basal consolidative opacities, more on the right side. CT: computed tomography

Computed tomography (CT) angiography revealed bilateral posterior-basal consolidative opacities, more on the right side as described; such a distribution is highly suggestive of aspiration pneumonia (Figure 3).

**Figure 4:**
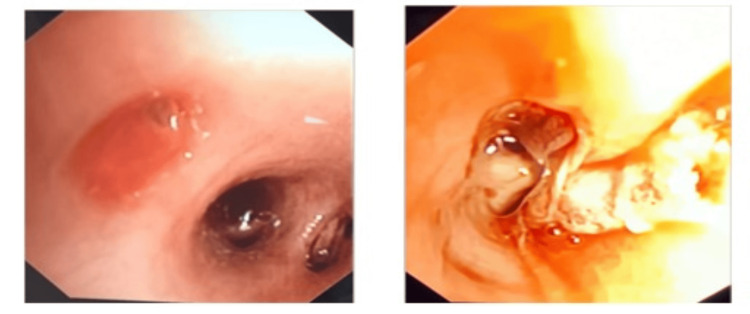
(A) Left: bronchoscope inserted into the posterior segment of the right lower lobe. (B) Right: localized pulmonary hemorrhage from the posterior segment of the basal segment of the lower lobe visualized in bronchoscopy.

The next day, the child was taken for bronchoscopy under general anesthesia. Flexible bronchoscope 4.8 was introduced easily through a laryngeal mask airway (LMA). The larynx was normal with some amount of old blood. The trachea was visualized clearly and appeared normal. The left and right main bronchi have no malformation; no bleeding was visualized. Once the scope was advanced in the right lung, the upper lobe revealed no abnormality; however, it was noted that the intermediate bronchus was blocked by a huge clot (Figure 4B), which was removed after multiple attempts with bronchoalveolar lavage (BAL). After retrieving the clot, there was no bleeding. Entrance to the right lower lobe was smooth, with no bleeding in the superior segment. The entrance of the common division of the lower lobe was normal. Basal segments revealed fresh blood gushing from the posterior basal segment, treated by multiple injections of adrenaline 1/100000 (of 10 ml each), after which the bleeding was controlled. 

The patient was re-intubated and shifted to the pediatric intensive care unit (PICU).

Bronchoscopy concluded localized pulmonary hemorrhage from the posterior segment of the basal segment of the lower lobe, consistent with vascular or pulmonary malformation.

He was seen by a cardiologist and cardiac intensivist, after which a decision to perform cardiac catheterization was made. Following this, an echocardiography was performed, which showed normal cardiac anatomy. 

**Figure 5:**
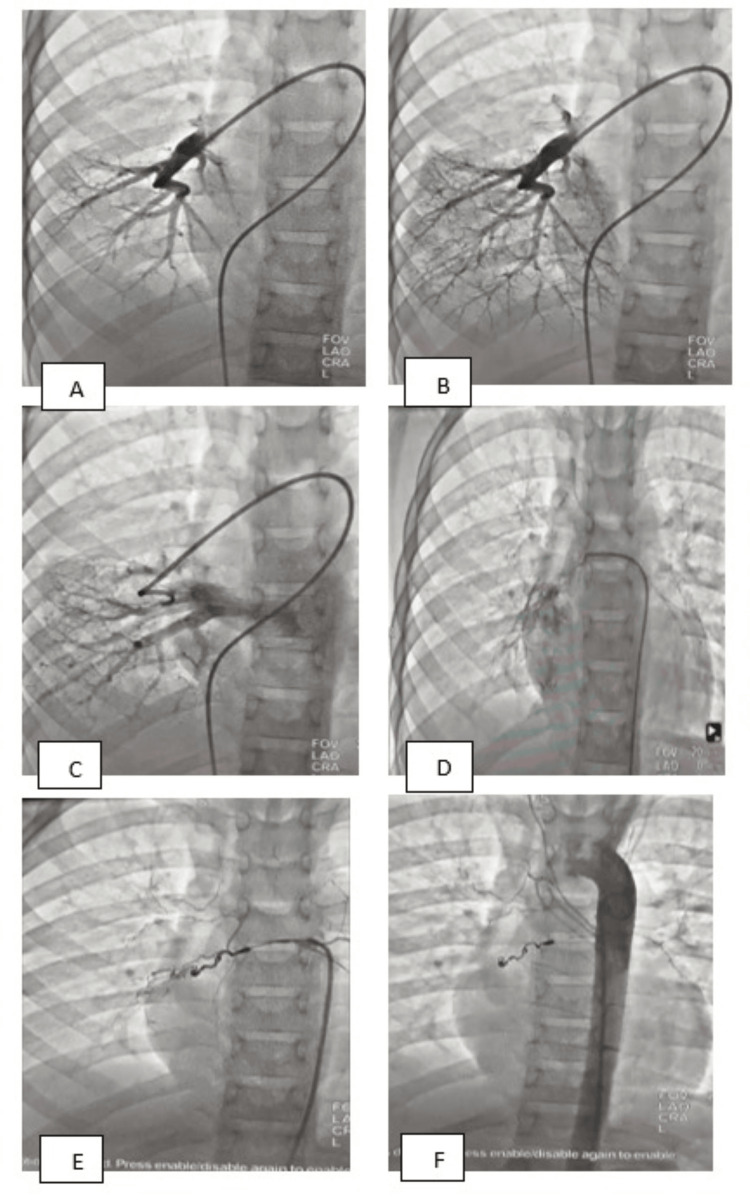
Angiogram with cardiac catheterization. (A) Through the sheaths, pulmonary and aortic catheterization and angiograms were performed. The 5F pigtail catheter was advanced to the left and right pulmonary artery from the venous side using Terumo wire. Selective right lower lobe and right and left pulmonary angiographies were done in the lateral and AP positions. (B and C) The angiogram showed normal pulmonary vascularization and normal pulmonary venous return to the left atrium. The 5F pigtail catheter was advanced to the ascending aorta retrogradely and aortic cineangiogram taken in the lateral and AP positions. (D) The angiogram showed normal aortic root with normal coronaries and cranial branches of the aorta; however, a small collateral artery was seen arising from the anterior part of thoracic aorta, and selective angiogram was taken which showed two branches supplying blood to the right upper and lower posterior lobes of the lung in connection with pulmonary arteries. The return of contrast to the left atrium via pulmonary veins was observed. (E) The 5F pigtail catheter and coronary guidewire were used to enter the bronchial artery, and then the guidewire was taken back, after which the occlusion was performed using a 2×4 mm and 3×8 mm Azur CX18 coil attached to the 2.7F Progreat delivery system. (F) The devices were successfully released, and a repeat fluoroscopy showed they were well-seated and there was no residual flow. AP: anteroposterior

The cardiac catheterization showed a small collateral artery arising from the anterior part of the thoracic aorta, and a selective angiogram was taken, which showed two branches supplying blood to the right upper and lower posterior lobes of the lung in connection with pulmonary arteries (Figure 5). The return of contrast to the left atrium via the pulmonary veins was observed. Pulmonologists' and interventional radiologists' opinions were taken for the closure of this abnormal vascular structure or bronchial artery, and they agreed to close it with the micro coil.

After a day of observation in the PICU post-procedure, the patient was transferred to the general pediatric ward, where he remained vitally and clinically stable, with no further complaints.

The above case was a nine-year-old previously healthy boy who was diagnosed with pulmonary arterio-arterial malformation and who presented with hemoptysis due to bleeding into the lungs that required the occlusion of the bronchial artery via cardiac catheterization.

Multiple investigations to identify the systemic causes of pulmonary hemorrhage were performed, including ANA, double-stranded DNA antibody, rheumatoid factor quantitative index (RF QI), endomysial antibody, c-ANCA, P-ANCA, antiphospholipid immunoglobulin M (APL IgM), anti-GBM IgG, and anti-tissue transglutaminase (anti-tTG) IgA, which were all negative.

## Discussion

Arterio-arterial malformations can cause vague respiratory symptoms like shortness of breath or chest pain, but may also present with severe signs such as hemoptysis, which is rare but dangerous in children due to the fragility of abnormal vessels. Hematemesis is unusual in these cases and often occurs when young children swallow blood from hemoptysis rather than expectorate it. Although most published reports focus on arteriovenous malformations, arterio-arterial malformations have been described in both children and adults, particularly in the brain, lungs, and coronary arteries [7]. In adults, they are usually linked to atherosclerosis, trauma, medical interventions, or previous vascular surgery, while pediatric cases may be associated with congenital vascular disorders, developmental anomalies, or HHT, though many remain idiopathic until symptoms or imaging reveals them [8]. This case highlights the need to include rare vascular malformations in the differential diagnosis of GI and respiratory bleeding in children.

Literature review

In a case published in a UK-based journal, a previously healthy nine-year-old girl presented with sudden-onset massive hemoptysis. Imaging via contrast-enhanced CT angiography revealed an unusual broncho-pulmonary arterial malformation. Despite the absence of underlying pulmonary or cardiac disease, this anomaly caused life-threatening bleeding. The patient was successfully treated with transcatheter embolization. Her symptoms resolved temporarily. However, she experienced a recurrence of hemoptysis several months later, prompting repeat imaging, which revealed persistent or recurrent abnormal vascular communication. A second embolization procedure was performed, after which she remained symptom-free during follow-up [6,9].

In another case report, a 12-year-old girl had two episodes of massive hemoptysis. Initial tests, including bronchoscopy and CT angiography, showed signs of bleeding, but they could not find the exact cause. It was only after doing catheter-based angiography that doctors discovered a fistula between the bronchial artery and the pulmonary artery. The abnormal connection was successfully treated with embolization [10].

A noteworthy case involved an 11-year-old boy who initially presented with mild hemoptysis and pneumonia but rapidly developed massive bleeding. Bronchial arteriography revealed a malformation consisting of a bronchial artery hypertrophy, a bronchial-pulmonary artery fistula, and an ectopic bronchial artery originating from the renal artery. The patient was successfully treated with bronchial artery embolization and bronchoscopy, resulting in full recovery [11].

A reported case involved a nine-month-old boy who presented with massive hemoptysis, diagnosed with bronchial Dieulafoy's disease (BDD). BDD is a rare vascular malformation characterized by a persistently dilated bronchial artery and is especially uncommon in infants. He was successfully treated with bronchial artery embolization, with no recurrence at the one-year follow-up [12].

A retrospective study analyzing CT and angiographic data from 74 children with pulmonary hemorrhage found that 30 cases had bronchial artery-pulmonary artery fistulas (BA-PAF), with a strong tendency for lesions to localize in the right middle and lower lobes. The most common CT findings included bronchial artery tortuosity and thickening, with or without visible consolidation. Notably, 41.2% of children with BA-PAF had normal chest CT findings, while others showed unilateral changes or ground-glass opacities. These results highlight that in children with hemoptysis and either subtle or absent CT abnormalities, primary BA-PAF should still be considered, especially if the right middle lobe is involved [13].

## Conclusions

This case reminds us how challenging pediatric presentations can be. A previously healthy nine-year-old boy came in with what looked like a simple case of vomiting blood, but it turned out to be a pulmonary arterio-arterial malformation. Despite normal initial investigations, it was only through thorough evaluation, including bronchoscopy and cardiac catheterization, that the true cause was uncovered. Transcatheter coil embolization was successful, and the child recovered without further bleeding. This case emphasizes the importance of keeping rare diagnoses in mind, especially when common causes have been ruled out, and highlights the value of a multidisciplinary approach in managing complex pediatric cases.
